# Strongyloidosis Hyperinfection Syndrome in an HIV-Infected Patient: A Rare Manifestation of Immune Reconstitution Inflammatory Syndrome

**DOI:** 10.1155/2018/6870768

**Published:** 2018-10-28

**Authors:** Kartik Natrajan, Mahenderkumar Medisetty, Raviraj Gawali, Ajit Tambolkar, Divya Patel, Vinay Thorat, Nachiket Dubale, Vrushali Khirid, Chinmay Saraf, Ameet Dravid

**Affiliations:** ^1^Department of Medicine, Poona Hospital and Research Center, Pune, Maharashtra, India; ^2^Department of Pathology, Poona Hospital and Research Center, Pune, Maharashtra, India; ^3^Department of Gastroenterology, Poona Hospital and Research Center, Pune, Maharashtra, India; ^4^Department of Pulmonology, Poona Hospital and Research Center, Pune, Maharashtra, India; ^5^Precision Diagnostics and Biosciences, Pune, Maharashtra, India

## Abstract

Parasitic infections such as *Strongyloides stercoralis* and HIV have been reported to coexist, particularly in resource-limited settings such as India. In an immunocompromised host, *S. stercoralis* can progress to strongyloidiasis hyperinfection syndrome (SHS). However, SHS is not common in patients with advanced HIV disease. Immune reconstitution inflammatory syndrome (IRIS) developing after initiation of antiretroviral therapy (ART) can target multiple pathogens including *S*. *stercoralis*. The authors present here a 46-year-old HIV-infected female who was recently diagnosed with HIV-1 infection, started ART, and developed SHS. Her upper GI endoscopy revealed severe gastroduodenitis, and X-ray chest showed extensive bilateral pneumonitis. We could identify *S. stercoralis* in induced sputum and duodenal biopsy. We could also identify gut inflammation to restrict invading parasites. After receiving antihelminthic therapy, she showed improvement, a course of events that fit the diagnosis of unmasking *S. stercoralis* IRIS.

## 1. Background

*Strongyloides stercoralis* typically infects the small bowel (duodenum and jejunum) of human hosts and is endemic to tropical and subtropical regions. The helminth enters the body through the skin and initially migrates through the venous peripheral blood system to the lung parenchyma. It ascends the tracheobronchial tree and is swallowed to reach the small intestine. After initial infection, mature females in the duodenum produce rhabditiform larvae that can develop into infective filariform larvae and reinfect the host through penetration of intestinal mucosa. This unique autoinfective capability facilitates chronic infection in a single host for decades [1]. Most immunocompetent hosts are asymptomatic. Strongyloidiasis hyperinfection syndrome (SHS) is a manifestation of accelerated autoinfection in an immunocompromised host (chronic steroid users and HTLV-1-infected individuals) involving multiple organs (disseminated disease) and extensive gastrointestinal (GI) involvement. The massive dissemination of filariform larvae to the lungs, liver, heart, central nervous system, and endocrine glands induces inflammation that may result in symptomatic dysfunction of these organs and even septic shock. SHS is estimated to occur in 1.5%–2.5% of the patients with strongyloidiasis [2]. *S. stercoralis* remains a neglected tropical disease, and its association with HIV coinfection is sparsely studied in resource-limited settings such as India. It is reflected by the paucity of the published literature on HIV/*S. stercoralis* coinfection [3–5]. Since 2005, there have been reports of seven patients presenting with SHS after antiretroviral therapy (ART) initiation [6–12]. ART-associated immune reconstitution inflammatory syndrome (IRIS) to *S. stercoralis* may be responsible for it. We hereby report a case of SHS as a component of IRIS in an HIV-positive individual recently initiated on ART in Pune, India.

## 2. Case Report

**Figure 1:**
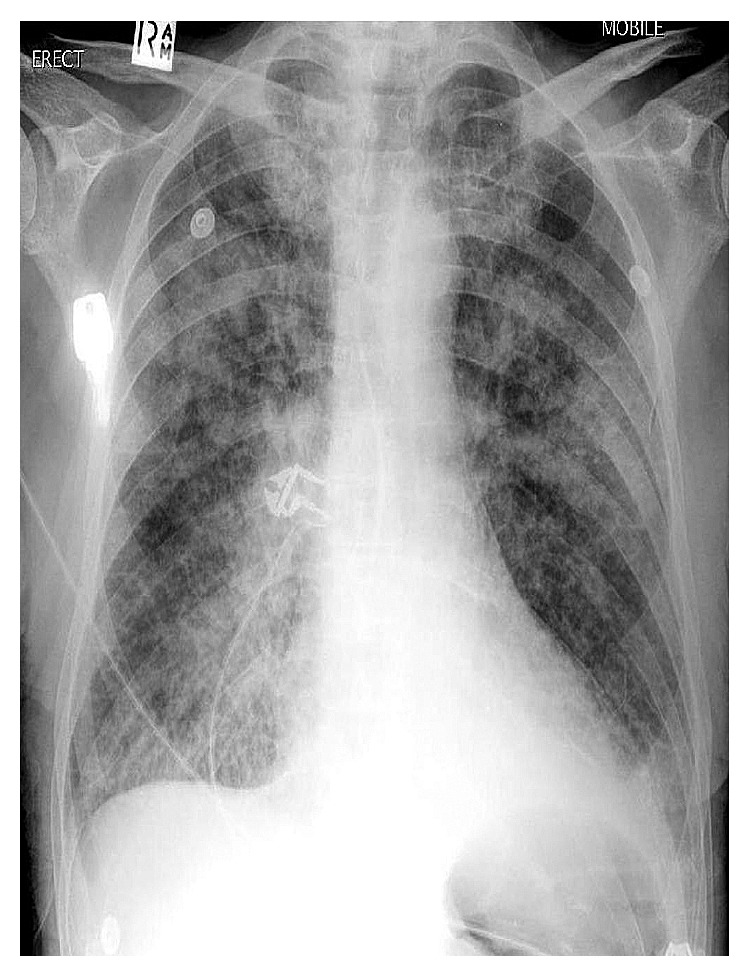
Chest X-ray of patient with SHS showing bilateral extensive pneumonic consolidation.

**Figure 2:**
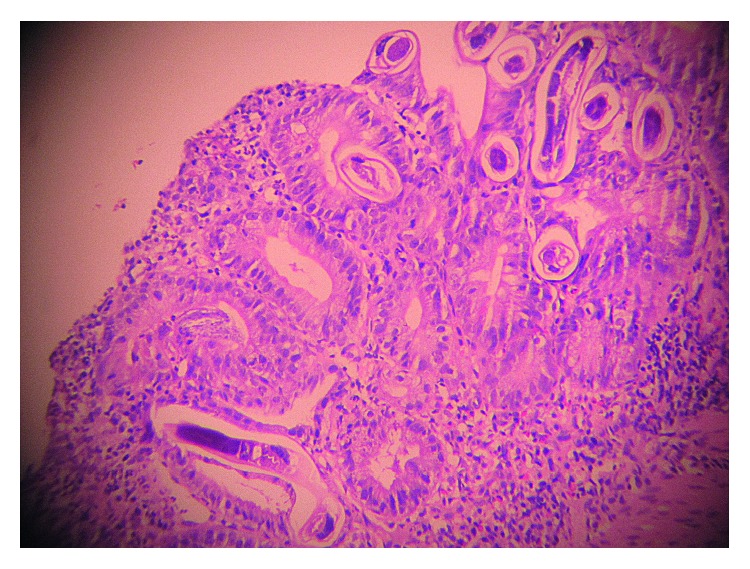
Duodenal histopathology showing gut inflammation with mucosal disruption.

**Figure 3:**
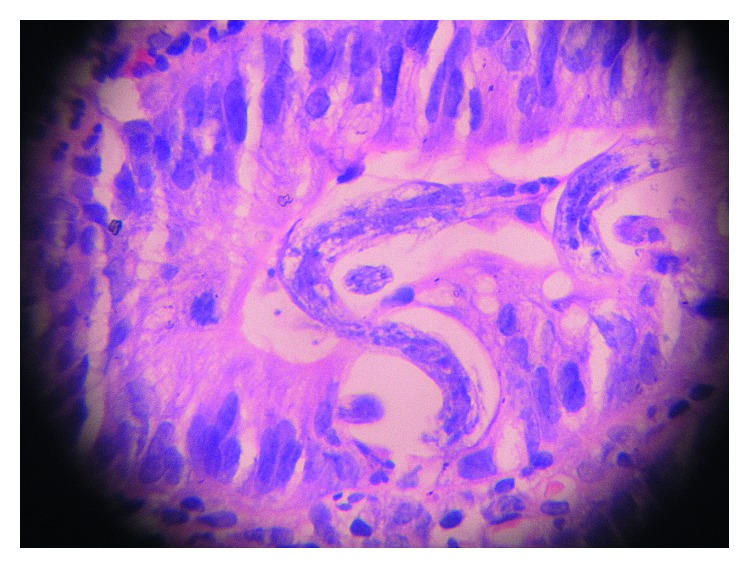
Duodenal histopathology showing eggs, larvae, and adult forms of *Strongyloides stercoralis*.

A 46-year-old female presented with complaints of fever, breathlessness on minimal exertion, vomiting, abdominal pain, and reduced appetite since 10 days. She also complained of weight loss of 10 kilograms in the last 6 months. She was diagnosed with HIV-1 infection, 1 month prior, and was prescribed tenofovir, lamivudine, and efavirenz fixed-dose combination single-pill regimen. Her baseline CD4 count was 68 cells/mm^3^, and plasma HIV-1 viral load was 867,000 copies/ml. She had no comorbidities or prior significant medical history. On examination, she was febrile (temperature: 100 degrees Fahrenheit) with pulse (100/min), blood pressure (110/60 mm Hg), and respiratory rate (24/min). Respiratory examination revealed crepitations in bilateral inframammary, infraaxillary, and infrascapular areas. There was diffuse abdominal tenderness but no organomegaly. Rest of the examination including fundoscopy was unremarkable. Hemoglobin was 8.3 g/dl while rest of the biochemical investigations was normal. CD4 count and plasma HIV-1 viral load after 1 month of ART was 190 cells/mm^3^ and 9,500 copies/ml, respectively, suggesting satisfactory immune reconstitution. The arterial blood gas (ABG) was suggestive of hypoxia (pO_2_-63 mm Hg) on room air. Chest X-ray was suggestive of bilateral, extensive, and patchy consolidation suggestive of infective etiology (Figure 1). Sonography of abdomen showed multiple mesenteric nodes with the largest size of 21 mm by 17 mm, grade 2 fatty liver, and dilated portal vein. The CT scan of chest revealed bilateral ground glass haziness suggestive of pneumocystis carinii pneumonia (PCP). CT abdomen showed biliary dilatation due to distal CBD stricture, mesenteric lymphadenopathy and mild diffuse thickening of the caecum, and ascending and transverse colon. 2D echocardiography of the heart was normal. Blood culture was sterile, and serum procalcitonin was normal. Keeping a provisional diagnosis of IRIS to *Pneumocystis jirovecii* or *Mycobacterium tuberculosis* in mind, she was started on high-dose trimethoprim-sulphamethoxazole (TMP-SMX), oral steroids, and empirical antitubercular therapy (ATT) in addition to ART. She had diarrhea on admission, which subsided in 2 days after taking loperamide. Stool routine and modified Ziehl–Neelsen (ZN) examination was unremarkable. Due to a suspicion of CMV enterocolitis, she was also started on empirical oral valgancyclovir. Bronchoscopy with BAL was attempted but had to be aborted in view of worsening hypoxia during procedure. For establishing diagnosis, upper GI endoscopy and colonoscopy were attempted. Upper GI endoscopy revealed diffuse gastroduodenitis. Colonoscopy showed patchy colitis. Duodenal biopsy revealed focal blunting, ulceration, and neutrophilic exudates (Figure 2). The surface epithelium showed several eggs, larvae, and adult forms of *S. stercoralis* (Figure 3). Lamina propria showed moderate mononuclear inflammatory infiltrate with lymphoid follicle formation and many admixed eosinophils (Figures 2 and 3). There was no evidence of tuberculosis or malignancy. Induced sputum also demonstrated larvae of *S. stercoralis*. As a result, diagnosis of SHS as a manifestation of IRIS was made, and she was started on an oral combination of albendazole (400 mg twice a day) and ivermectin (200 *µ*g/kg). Inspite of antihelminthic treatment, her dyspnea worsened. Her repeat ABG showed hypoxia (PO_2_ –47 mm Hg, Spo_2_ 89%) and respiratory alkalosis. She developed hypotension (blood pressure 80/60 mm Hg) and altered sensorium in the form of increasing drowsiness (Glasgow Coma Scale 12/15). She was shifted to the Intensive Care Unit (ICU) for the same. MRI brain and CSF examination were normal while serum sodium level had dropped to 125 mEq/L. Noradrenaline infusion and noninvasive BIPAP ventilation were started. She was initiated on intravenous (i.v.) meropenem, i.v. teicoplanin, and i.v. fluconazole in addition to antihelminthic agents, steroids (in tapering doses), and ART. ATT and valganciclovir were withdrawn while TMP-SMX was reduced to prophylactic dose. She gradually started improving over the next 7 days with improvement in sensorium and reduction in breathlessness. She was shifted out of the ICU by day 21 of admission. Her CXR showed complete resolution of pneumonic consolidation. She was discharged on oral antihelminthic treatment which she continued for another 8 weeks. Follow-up endoscopy studies showed complete clearance of *S. stercoralis*.

## 3. Discussion

Our case report is the eighth reported case of IRIS to *S. stercoralis* worldwide and the first from India. Advanced HIV has not been linked with disseminated strongyloidiasis despite a 21-fold increased risk of intestinal infection in endemic areas [3]. Few previous reports from India have shown that the prevalence of *S. stercoralis* in HIV-infected patients ranged from 0% to 27.3% [3]. However, cases of SHS have been rarely described in HIV-infected patients in India [4, 5]. The absence is explained by the fact that in HIV-positive patients with higher CD4^+^ cell counts, direct development of infective filariform larvae in the gut (autoinfection) is favored, compared with those with lesser immune function (low CD4 count or advanced HIV disease), in whom, indirect development is favored. Indirect development of filariform larvae in individuals with advanced HIV disease reduces the opportunity for dissemination to occur [13]. T helper cell type 2 (Th2) immune function is well preserved in advanced HIV which also prevents SHS or dissemination [13, 14]. As a result, disseminated strongyloidiasis in HIV was removed from the Center for Disease Control (CDC) and World Health Organization (WHO) lists of HIV specific infections in 1987. Since then, physician awareness of SHS in HIV has reduced [11]. This might change in future due to cases of SHS being reported after initiating ART, supporting the existence of immune reconstitution inflammatory syndrome(IRIS) towards *S. stercoralis* [6–12]. Two distinct patterns of IRIS are described in the literature and are now recognized as “paradoxical IRIS” and “unmasking IRIS.” In paradoxical forms of IRIS, symptoms and signs associated with a known opportunistic infection (OI), for which treatment is under way, recur or become acutely worse, despite an earlier favorable response to therapy prior to ART. In unmasking IRIS, a new OI presents with a pronounced inflammatory component following ART initiation [15]. Although *S. stercoralis* is listed as an immune reconstitution inflammatory syndrome- (IRIS-) related pathogen, this condition is scarcely reported in the literature [14]. In the seven previous reports of IRIS to *S*. *stercoralis*, unmasking IRIS was seen in five cases [6–10] while paradoxical IRIS was seen in the other two [11, 12]. IRIS was seen approximately 19–150 days after ART initiation.

In our patient, unmasking IRIS occurred 30 days after ART initiation and manifested as severe gastroduodenitis and patchy colitis leading to severe abdominal pain, vomiting, and intermittent diarrhea. In addition, pulmonary involvement was evidenced by dyspnea and bilateral pneumonitis on CXR leading to acute respiratory distress syndrome (ARDS). The presence of immune reconstitution was indicated by an increased CD4+ count and decreased viral load comparable to baseline. The temporal relationship between ART initiation and SHS in this case suggests a causal association between the two events. There are several possible explanations for this rapid clinical deterioration. First, a high parasite burden was present prior to ART, and the development of symptoms was a result of a renewed immune response to the pathogen that is typical of unmasking IRIS. Post-ART duodenal biopsy showed heavy parasite load coupled with moderate mononuclear infiltrate, eosinophil infiltration, and granuloma formation (Figures 2 and 3). Second, addition of steroids for treatment of IRIS could have also contributed to clinical deterioration during admission. However, our patient was not exposed to steroids prior to diagnosis of SHS, unlike other case reports [6, 9]. Our patient of SHS did not show eosinophilia on peripheral blood smear [6, 7] or haematogenous spread of gut coliforms [6]. Our histopathology findings were also not consistent with earlier case reports which showed no significant inflammatory response in the postmortem histopathology in patients with SHS after immune reconstitution [10, 11]. Temporary withdrawal of ART in view of life-threatening IRIS was not done unlike earlier instances [11]. We used antihelminthic drug regimens for extended duration as shown in previous cases of SHS. Our case is the eighth-reported patient with progressive strongyloidiasis after immune reconstitution, lending additional support for the recognition of IRIS to *S. stercoralis*. We believe that the prevalence of this phenomenon is much higher in resource-limited settings such as India which is coendemic to HIV and *S. stercoralis* and has seen rapid scale up of ART in recent years. However, lack of awareness of health care personnel or lack of facilities in resource-limited settings to establish diagnoses leads to underreporting [14].

## 4. Conclusions

In conclusion, physicians working in resource-limited settings such as India should consider parasitic infections as a differential diagnosis while evaluating cases of IRIS in HIV-infected patients starting ART.
